# The stratified effects of repetitive transcranial magnetic stimulation in upper limb motor impairment recovery after stroke: a meta-analysis

**DOI:** 10.3389/fneur.2024.1369836

**Published:** 2024-04-02

**Authors:** Ran Li, Sihan Liu, Tianyuan Li, Kun Yang, Xue Wang, Wenjiao Wang

**Affiliations:** ^1^Department of Rehabilitation Center, Fu Xing Hospital, Capital Medical University, Beijing, China; ^2^Capital Medical University Eighth Clinical School, Beijing, China; ^3^Department of Evidence-based Medicine, Xuan Wu Hospital, Capital Medical University, Beijing, China; ^4^Department of Medical Library, Xuan Wu Hospital, Capital Medical University, Beijing, China

**Keywords:** repetitive transcranial magnetic stimulation, stroke, upper limb, motor impairment, rehabilitation, improvement

## Abstract

**Background:**

The recovery of upper extremity motor impairment after stroke remains a challenging task. The clinical effectiveness of repetitive transcranial magnetic stimulation (rTMS), which is believed to aid in the recovery process, is still uncertain.

**Methods:**

A systematic search was conducted in Medline (Ovid), Cochrane and Embase electronic databases from March 28, 2014, to March 28, 2023. The inclusion criteria consisted of randomized controlled trials that assessed the effects of rTMS on the recovery of upper limb motor impairment among stroke patients. Various measurements, including the Fugl Meyer Assessment Upper Extremity Scale (FMA-UE), Brunnstrom recovery stage, Action Research Arm Test (ARAT), and Barthel index, were evaluated both before and after the intervention.

**Results:**

Nineteen articles with 865 patients were included. When considering only the rTMS parameters, both inhibitory and excitatory rTMS improved FMA-UE (MD = 1.87, 95% CI = [0.88]–[2.86], *p* < 0.001) and Barthel index (MD = 9.73, 95% CI = [4.57]–[14.89], *p* < 0.001). When considering only the severity of upper limb hemiplegia, both less severe (MD = 1.56, 95% CI = [0.64]–[2.49], *p* < 0.001) and severe (MD = 2.05, 95% CI = [1.09]–[3.00], *p* < 0.001) hemiplegia benefited from rTMS based on FMA-UE. However, when considering the rTMS parameters, severity of hemiplegia and stroke stages simultaneously, inhibitory rTMS was found to be significantly effective for less severe hemiplegia in the acute and subacute phases (MD = 4.55, 95% CI = [2.49]–[6.60], *p* < 0.001), but not in the chronic phase based on FMA-UE. For severe hemiplegia, inhibitory rTMS was not significantly effective in the acute and subacute phases, but significantly effective in the chronic phase (MD = 2.10, 95% CI = [0.75]–[3.45], *p* = 0.002) based on FMA-UE. Excitatory rTMS was found to be significantly effective for less severe hemiplegia in the acute and subacute phases (MD = 1.93, 95% CI = [0.58]–[3.28], *p* = 0.005) based on FMA-UE. The improvements in Brunnstrom recovery stage and ARAT need further research.

**Conclusion:**

The effectiveness of rTMS depends on its parameters, severity of hemiplegia, and stroke stages. It is important to consider all these factors together, as any single grouping method is incomplete.

## 1 Introduction

Motor weakness is the most common disability after stroke. While two-thirds of patients can walk independently, less than half can restore basic upper limb function 1 year after stroke, which severely limits their independence (1). Since cortical reorganization is essential for motor improvement (2), it is crucial to modulate cortical excitability using appropriate technologies.

Noninvasive brain stimulation can modify cortical excitability and improve behavioral performance by regulating brain electrical activity. Several studies have also suggested the effectiveness of combining noninvasive brain stimulation with other therapies to enhance upper limb motor impairment (3–5). According to estimates, the application of repetitive transcranial magnetic stimulation (rTMS) could potentially lead to a 10–20% improvement in upper limb motor function after five sessions (6). However, it is not uncommon to find inconsistencies in the effectiveness of rTMS. Some literature supports the effectiveness of rTMS (7–9), while others suggest that it is ineffective (10, 11). These mixed outcomes highlight the need for further exploration of the clinical application parameters (12).

According to the interhemispheric inhibition theory, it is recommended to apply inhibitory stimuli to the contralesional hemisphere and excitatory stimuli to the ipsilesional hemisphere (2). Which kind of stimulation is more effective? Xia et al. (13) suggested that the excitatory high-frequency rTMS was the primary stimulation protocol. However, contrary to this, another study found that the inhibitory low-frequency rTMS implementation could induce the highest recovery changes in different areas depending on the severity of hemiplegia (14). For severe hemiplegia, the shoulder and elbow showed the highest recovery changes, while for moderate hemiplegia, the wrist and finger showed the highest recovery changes, and coordination showed the highest recovery changes for mild hemiplegia (14). These studies suggest that there may be other confounding factors that have not yet been identified.

The severity of upper limb hemiplegia is an important factor that should be taken into account. Different motor deficits have distinct recovery patterns (15). The proportional recovery rule varies between mild-to-moderate paresis and severe paresis (16). The rehabilitation methods for mild-to-moderate hemiplegia have been recognized by most therapists, while those for severe hemiplegia lack treatment consistency until now (17). Therefore, when considering the rTMS stimulus parameters, it is also important to consider the characteristics of the patients themselves. The severity of hemiplegia is one such characteristic that cannot be ignored. Different brain injuries will initiate distinct brain reorganizations. It is reasonable to expect the same rTMS stimulation parameters to have varying effects among different degrees of hemiplegia (14).

The effect of rTMS is also influenced by the stage of stroke. In a longitudinal study using functional near-infrared spectroscopy, it was observed that there is a progressive shift in cortical activity lateralization from bilateral to ipsilesional patterns within 3 months after stroke. This shift is accompanied by an increase in the Fugl-Meyer score (18). Another study using resting-state fMRI showed a decrease in the lateralization index initially, followed by an increase from <7 to 180 days after stroke onset (19). It is reasonable to expect that different brain functional states at different stages of stroke will lead to different responses to rTMS.

The recovery of upper limb function after stroke is a complex process. In order to accurately measure and specify the effectiveness of rTMS, it is important to consider various factors such as the severity of hemiplegia, the stage of stroke, and the parameters of rTMS. However, there is currently no comprehensive meta-analysis that incorporates all three of these influencing factors simultaneously. Therefore, our study aims to conduct a stratified analysis to investigate and clarify the role of rTMS in the recovery of upper limb function after stroke.

## 2 Materials and methods

We adhered to the Preferred Reporting Items for Systematic Reviews and Meta-Analyses statement while reporting our findings. Our Meta-analysis has been registered at PROSPERO (CRD42023420797).

### 2.1 Literature search strategy

To find relevant studies, we used the following Mesh terms or keywords: “Stroke,” “Upper limb,” “Hemiplegia,” and “Transcranial Magnetic Stimulation.” We conducted a systematic search of the Medline (Ovid), Cochrane, and Embase electronic databases for studies published in English from March 28, 2014, to March 28, 2023. Before starting the research, all authors agreed on the search strategy. Additionally, we manually checked the reference lists of included studies and relevant systematic reviews/meta-analyses to identify any other studies that may have been missed during the database search.

### 2.2 Eligibility criteria

Studies meeting the inclusion criteria will be included: (1) Participants: stroke patients with upper limb motor impairment; (2) Intervention: the intervention group received rTMS stimulation with or without other therapies; (3) Comparison: the control group received other therapies with or without sham rTMS; (4) Outcomes: FMA-UE, Brunnstrom recovery stage, ARAT and Barthel index; and (5) Study designs: randomized controlled trials.

Studies will be excluded if they meet any of the following criteria: (1) Replicated articles; (2) Non-adult stroke patients; (3) Intervention was implicated only one session; (4) Clinical registration trials; (5) Protocol studies; (6) Reviews, systematic reviews, meta-analyses; (7) Case reports; (8) Conference abstracts; (9) Outcome measures were not reported; and (10) Outcome measures were reported but without extractable format.

### 2.3 Study selection

All articles searched from the database were imported into the Endnote software. Two independent reviewers screened and assessed the relevance of the articles. Firstly, the duplicated articles were removed. Then, the remaining articles were excluded based on the information provided in the title and abstract. In cases where there was no consensus or the title and abstract did not provide sufficient information, the full-text of the articles was thoroughly reviewed. Conflicts between reviewers were resolved by carefully examining each article against all the inclusion criteria and engaging in necessary discussions.

### 2.4 Outcome measure of interest

Among the motor assessments for upper limb after stroke, the FMA-UE and ARAT are the first two recommended scales (20). The FMA-UE is derived from the Brunnstrom recovery stage. The Barthel index and Functional Independence Measure are commonly utilized tools for assessing activities of daily life. Barthel index is more commonly used, while the Functional Independence Measure is considered more suitable for evaluating patients with severe stroke. In this study, the primary outcome measure was the FMA-UE score or Brunnstrom recovery stage, which represents the body function level; the secondary outcome measure was the ARAT score, which represents the activity level; the third outcome measure was the Barthel Index, which represents activities of daily life.

The FMA-UE consists of 33 items. The first item assesses reflex activity, with a score of 0 for no reflex activity and 2 for elicited reflex activity. The remaining items are scored on a three-point ordinal scale: 0 for inability to perform, 1 for partial performance, and 2 for flawless performance. The total score on the FMA-UE is 66 points, with higher scores indicating better motor performance. The severity of upper limb motor impairment can be determined by the FMA-UE score, with a range of 0–19 indicating severe impairment, 20–47 indicating moderate impairment, and 48–66 indicating mild impairment (21). Scores of 0–19 are classified as severe hemiplegia, while scores of 20–66 are classified as less severe hemiplegia.

### 2.5 Data extraction

A pre-determined template was used by the corresponding author to collect data. Two independent reviewers (RL and SL) performed the data extraction. Any differences between the reviewers were resolved through discussion with the corresponding author. The data extraction process included gathering information about the study (the first author, publication year, and study location), participant characteristics (the number of participants, stroke stages, and severity of upper limb motor impairment), details of the intervention group (including the type of intervention, rTMS stimulation intensity, rTMS stimulation site, and rTMS sessions), details of the control group (including the control condition, therapy dosage, and therapy sessions), and the outcome measurements.

### 2.6 Risk of bias assessment

The data extracted from the included studies was transferred into the Review Manager 5.4.1 software. Two reviewers assessed the risk of bias using the recommended tool for randomized trials from the Cochrane Collaboration (22). Any discrepancies were resolved through discussion. The risk of bias tool covers six domains: selection bias (random sequence generation, allocation concealment), performance bias (blinding of participants and personnel), detection bias (blinding of outcome assessment), attrition bias (incomplete outcome data), reporting bias (selective reporting), and other bias (anything else, ideally prespecified). The included studies were evaluated for low, unclear, or high risk of bias.

### 2.7 Data synthesis and statistical analysis

All analyses were performed using the Review Manager 5 software. Clinical heterogeneity among the included studies was assessed with the *I*^2^ statistic. A value ≥50% indicating a significant heterogeneity between studies, in response to which a random effect model was used to fulfill data synthesis. On the contrary, a fixed effect model was used in cases where *I*^2^ statistics value <50%.

If the change scores of the intervention and control groups between baseline and the time of intervention completion were provided, they were directly used for statistical analysis. Data presented with 95% CI or standard errors of mean, minimum and maximum, or quartiles were converted into means and standard deviations using Cochrane’s RevMan Calculator for Microsoft Excel (23). The change values were obtained from means and SDs using the formulae set given by Wan et al. (24). The outcome measures were continuous variables. The combined outcomes were calculated using the MD for means with small differences or the SMD for means with significant differences. The effect sizes and 95% CI were presented on forest plots. A *p* value <0.05 was considered statistically significant.

The publication bias of these studies was evaluated by visually measuring the symmetry of funnel plots.

## 3 Results

### 3.1 Study selection

**Figure 1:**
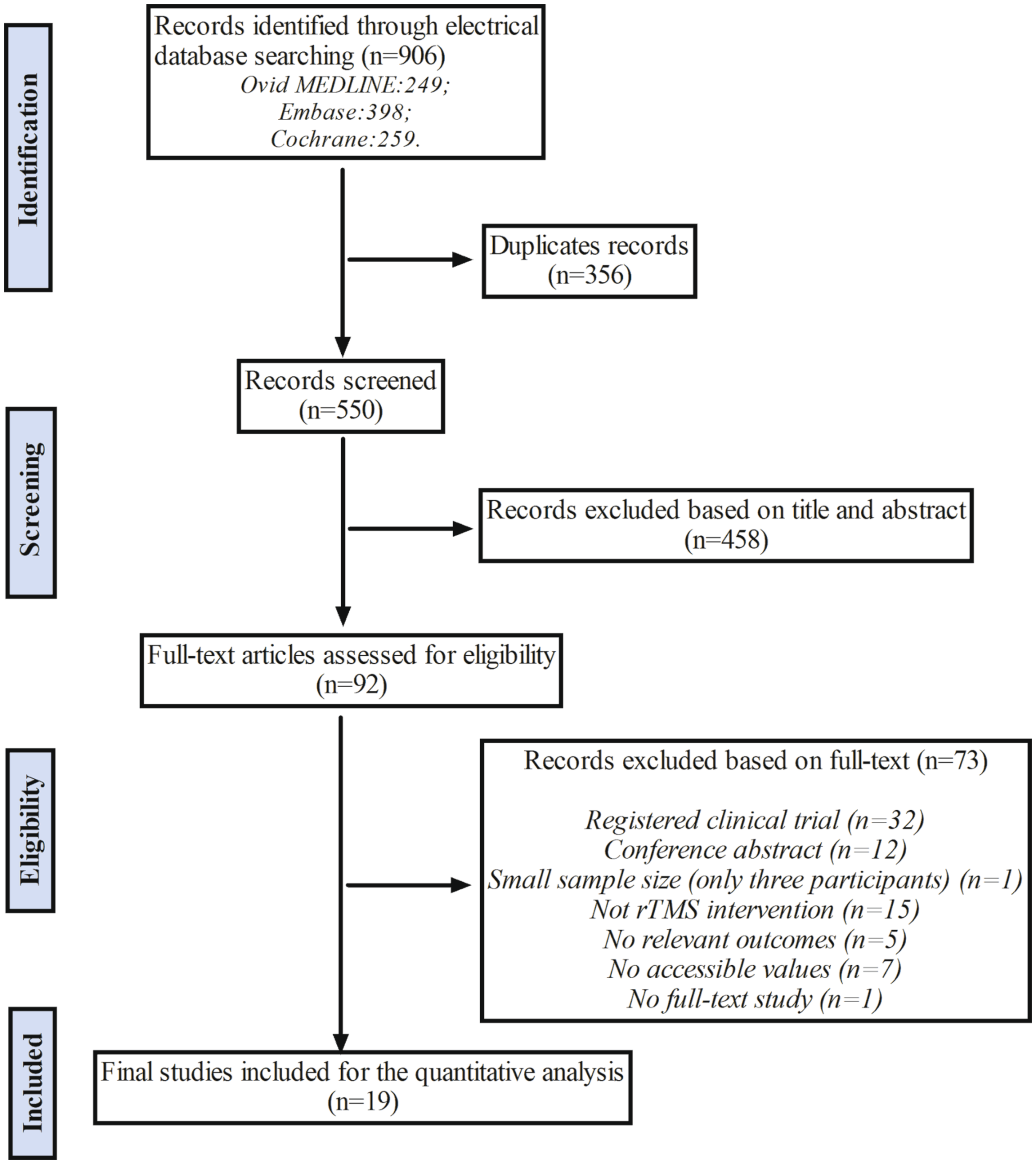
The flow chart of study selection. The rTMS indicates repetitive Transcranial Magnetic Stimulation.

A total of 906 relevant studies were identified from three electrical databases (249 from Ovid MEDLINE, 398 from Embase, and 259 from Cochrane). After removing 356 duplicated records, 550 records were screened based on titles and abstracts. Among them, 458 articles were excluded due to inappropriate study type, participants not fitting the criteria, or interventions unrelated to the theme of our study. Consequently, 92 studies met the inclusion criteria and underwent a thorough full-text review for eligibility. Among these 92 studies, 32 were excluded due to being registered clinical trials, 12 due to being conference abstracts, 1 due to a small sample size, 15 due to not being appropriate rTMS interventions, 5 due to not containing relevant outcomes, 7 due to not having accessible values, and 1 due to not being a full-text study. Therefore, a total of 19 studies were considered eligible for the quantitative analysis. The process is shown in the flow chart (Figure 1).

### 3.2 Characteristics of included studies

The characteristics of the 19 included articles are summarized in Table 1. All of the articles were randomized controlled trials. Geographically, 15 studies were conducted in Asia (7, 8, 11, 26–31, 34–39), three in the United States (10, 32, 33), and one in New Zealand (25). The sample sizes ranged from 12 (32) to 199 (10). Two articles focused on acute stroke (28, 34), seven articles focused on subacute stroke (7, 8, 29–31, 36, 38), one article focused on both acute and subacute stroke (39), six articles focused on chronic stroke (25–27, 33, 35, 37), and the remaining three articles focused on acute, subacute, and chronic stroke (10, 11, 32). Among the articles, 10 focused on less severe hemiplegia (7, 8, 10, 27–31, 33, 39), four focused on severe hemiplegia (26, 35, 36, 38), four did not mention the severity of hemiplegia (11, 25, 34, 37), and one article focused on both severe and less severe hemiplegia (32).

**Table 1:** Study characteristics of included studies.

References	Study location	No. of participants (Intervention/Control)	Stroke stages	Severity of upper limb motor impairment	Intervention group				Control group		Outcome measurements	
					Type of intervention	rTMS stimulation intensity	rTMS stimulation site	rTMS sessions	Control condition	Therapy dosage	Therapy sessions	
Ackerley et al. (25)	New Zealand	18 (9/9)	Chronic stroke	Unclear	iTBS + physical therapy	iTBS, 90% AMT, and 600 pulses	M1, affected hemisphere	10 sessions	Sham iTBS + physical therapy	45 min	10 sessions	ARAT
Aşkın et al. (26)	Turkey	40 (20/20)	Chronic ischemic stroke	Severe hemiplegia	rTMS + physical therapy	1 Hz, 90% RMT, and 1,200 pulses	M1, unaffected hemisphere	10 sessions	Physical therapy	Not mentioned	20 sessions	FMA-UE, Brunnstrom recovery stage
Chang et al. (11)	Taipei	31 (16/15)	Subacute and chronic stroke	Unclear	iTBS + physical therapy	iTBS, 80% RMT, and 600 pulses	M1, affected hemisphere	10 sessions	Sham iTBS + physical therapy	Not mentioned	10 sessions	FMA-UE, Brunnstrom recovery stage
Etoh et al. (27)	Japan	18 (9/9)	Chronic stroke	Less severe hemiplegia	rTMS + NMES, DAVS group	1 Hz, 90% RMT, and 600 pulses	M1, unaffected hemisphere	20 sessions	NMES, DAVS group	40 min	20 sessions	FMA-UE, ARAT
Guan et al. (28)	China	42 (21/21)	Acute stroke	Less severe hemiplegia	rTMS	5 Hz, 120% RMT, and 1,000 pulses	M1, affected hemisphere	10 sessions	Sham rTMS	Not mentioned	10 sessions	FMA-UE, Barthel index
Moslemi Haghighi et al. (29)	Iran	20 (10/10)	Subacute stroke	Less severe hemiplegia	rTMS + physical therapy	20 Hz, 90% RMT, and 2,000 pulses	M1, affected hemisphere	10 sessions	Physical therapy	Not mentioned	10 sessions	FMA-UE
Harvey et al. (10)	United States	199 (132/67)	Chronic stroke	Less severe hemiplegia	rTMS + rehabilitation therapy	1 Hz, RMT-not mentioned	M1, unaffected hemisphere	18 sessions	Sham rTMS + rehabilitation therapy	60 min	18 sessions	FMA-UE, ARAT
						Pulse-not mentioned						
Hosomi et al. (30)	Japan	42 (18/21)	Subacute stroke	Less severe hemiplegia	rTMS + rehabilitation therapy	5HZ, 90% RMT, and 500 pulses	M1, affected hemisphere	10 sessions	Sham rTMS + rehabilitation therapy	60 min	10 sessions	FMA-UE, Brunnstrom recovery stage
Long et al. (8)	China	42 (21/21)	Subacute stroke	Less severe hemiplegia	LF rTMS + rehabilitation therapy	1 Hz, 90% RMT, and 1,000 pulses	M1, unaffected hemisphere	15 sessions	Sham rTMS + rehabilitation therapy	Not mentioned	15 sessions	FMA-UE
Long et al. (8)	China	42 (21/21)	Subacute stroke	Less severe hemiplegia	LF-HF rTMS + rehabilitation therapy	10 Hz, 90% RMT, and 2,000 pulses	M1 of unaffected hemisphere for LF-rTMS; M1 of affected hemisphere for HF-rTMS	15 sessions	LF rTMS + rehabilitation therapy	1 Hz, 90% RMT, and 1,000 pulses	15 sessions	FMA-UE
Luk et al. (31)	China	24 (12/12)	Subacute stroke	Less severe hemiplegia	rTMS + rehabilitation therapy	1 Hz, 90% RMT, and 1,200 pulses	M1, unaffected hemisphere	10 sessions	Sham rTMS + rehabilitation therapy	30 min	30 sessions	FMA-UE, ARAT
Bonin Pinto et al. (32)	United States	18 (9/9)	Acute, subacute and chronic stroke	Severe and less severe hemiplegia	rTMS + fluoxetine	1 Hz, 100% RMT, and 1,200 pulses	M1, unaffected hemisphere	10 sessions	sham rTMS + fluoxetine	Not mentioned	1 month of drug treatment	FMA-UE
Rose et al. (33)	United States	19 (9/10)	Chronic stroke	Less severe hemiplegia	rTMS + rehabilitation therapy	1 Hz, 100% RMT, and 1,200 pulses	M1, unaffected hemisphere	16 sessions	Sham rTMS + rehabilitation therapy	60 min	16 sessions	FMA-UE, ARAT
Sasaki et al. (34)	Japan	58 (27/31)	Acute stroke	Unclear	BL-rTMS	LF-rTMS (1 Hz, 90% RMT, and 1,100 pulses)	M1 of unaffected hemisphere for LF-rTMS; M1 of affected hemisphere for HF-rTMS	5 sessions	HF-rTMS	10 Hz, 90% RMT, and 1,000 pulses	5 sessions	Brunnstrom recovery stage
						HF-rTMS (10 Hz, 90% RMT, and 1,000 pulses)						
Tosun et al. (7)	Turkey	18 (9/9)	Subacute stroke	Less severe hemiplegia	rTMS + rehabilitation therapy	1 Hz, 90% RMT, and 1,200 pulses	M1, unaffected hemisphere	10 sessions	Rehabilitation therapy	Not mentioned	20 sessions	FMA-UE, Brunnstrom recovery stage, and Barthel index
Motamed Vaziri et al. (35)	Iran	12 (6/6)	Chronic stroke	Severe hemiplegia	rTMS+ rehabilitation therapy	1 Hz, 60–80% RMT, and 1,200 pulses	M1, unaffected hemisphere	10 sessions	Sham rTMS + rehabilitation therapy	40 min	10 sessions	FMA-UE, Barthel index
Wang et al. (36)	China	30 (15/15)	Subacute stroke	Severe hemiplegia	rTMS+ rehabilitation therapy	10 Hz, 100% RMT, and 1,000 pulses	M1, unaffected hemisphere	14 sessions	Sham rTMS + rehabilitation therapy	40 min	14 sessions	Barthel index
Wu et al. (37)	China	105 (53/52)	Chronic stroke	Unclear	rTMS + rehabilitation therapy	1 Hz, 90% RMT, and 1,000 pulses	SMA and PMC, unaffected hemisphere	14 sessions	Rehabilitation therapy	30 min	14 sessions	Brunnstrom recovery stage
Zhao et al. (38)	China	17 (8/9)	Subacute stroke	Severe hemiplegia	rTMS +rehabilitation therapy + scalp acupuncture	1 Hz, 70% RMT, and 1,200 pulses	M1, unaffected hemisphere	14 sessions	Rehabilitation therapy + scalp acupuncture	Not mentioned	14 sessions	FMA-UE, Barthel index
Zheng et al. (39)	China	112 (58/54)	Acute and subacute stroke	Less severe	rTMS + virtual reality	1 Hz, 90% RMT, and 1,800 pulses	M1, unaffected hemisphere	24 sessions	Sham rTMS + virtual reality	30 min	24 sessions	Barthel index

rTMS, repetitive Transcranial Magnetic Stimulation; iTBS, intermittent Theta Burst Stimulation; AMT, Active motor threshold; M1, Primary motor cortex; ARAT, Action research arm test; FMA-UE, Fugl-Meyer assessment upper extremity scale; NMES, Neuromuscular electrical stimulation; DAVS, Direct application of vibratory stimulation; LF-rTMS, Low-frequency rTMS; HF-rTMS, High-frequency rTMS; BL-rTMS, Bilateral rTMS; SMA, Supplementary motor area; and PMC, Premotor cortex.

Eleven articles focused on inhibitory rTMS (7, 10, 26, 27, 31–33, 35, 37–39), six articles focused on excitatory rTMS (11, 25, 28–30, 36), and one article focused on both inhibitory and excitatory rTMS (8). Additionally, one article specifically focused on bilateral rTMS (34). The excitatory rTMS interventions could be either iTBS (11, 25) or rTMS. The stimulation frequency of rTMS was 5 Hz (28, 30), 10 Hz (8, 36), or 20 Hz (29). However, the stimulation frequency used for inhibitory rTMS was set at 1 Hz. The stimulation location for excitatory rTMS was primary motor cortex (M1) of the affected hemisphere, while for inhibitory rTMS was M1 of the unaffected hemisphere, except for one article that focused on inhibitory rTMS with supplementary motor area (SMA) and premotor cortex (PMC) regions of the unaffected hemisphere (37). Ten articles selected a 10-session protocol (7, 11, 25, 26, 28–32, 35); three articles selected a 14-session protocol (36–38); and the remaining articles involved protocols of 24 sessions (39), 20 sessions (27), 18 sessions (10), 16 sessions (33), 15 sessions (8), and five sessions (34). The control intervention in the studies was therapy with or without sham rTMS.

### 3.3 Risk of bias

**Figure 2:**
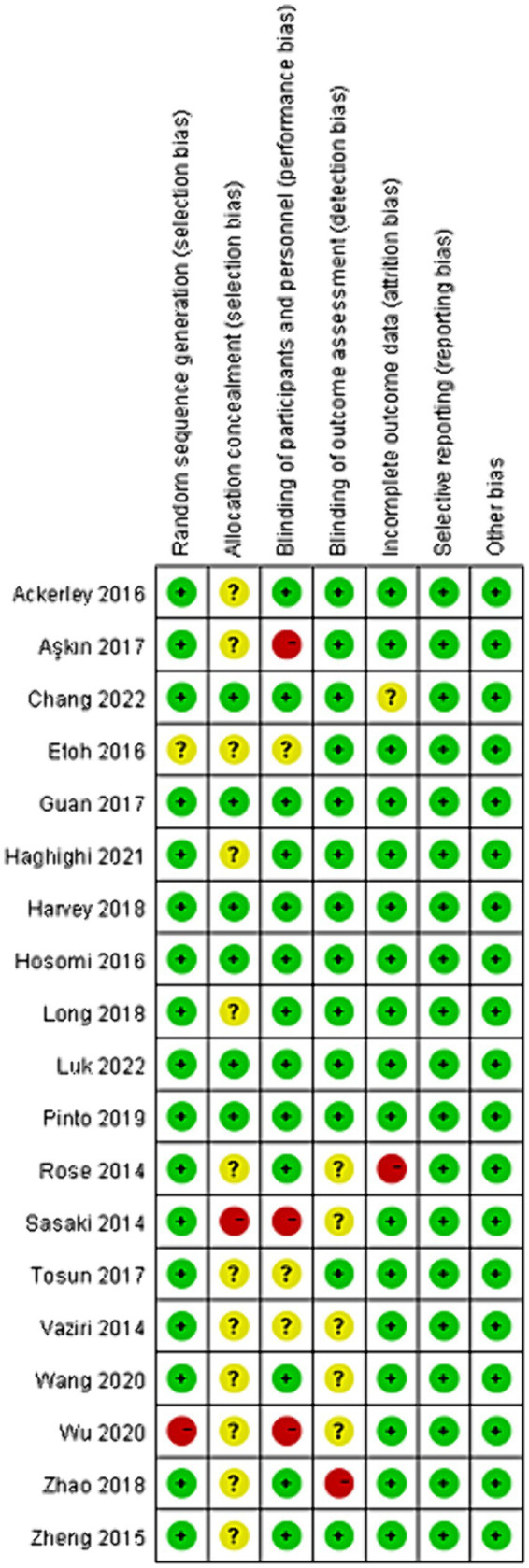
Risk of bias in individual studies.

In total, five out of 19 articles were classified as having a “low risk of bias” for all seven items recommended by the Cochrane Collaboration. Additionally, five out of 19 articles reported only one of the seven items. When evaluating each item individually, 17 out of 19 articles reported random sequence generation, six out of 19 articles reported allocation concealment, 13 out of 19 articles reported blinding of participants and personnel, and 13 out of 19 articles reported blinding of outcome assessment. Furthermore, 17 out of 19 articles reported no incomplete outcome data, and all articles reported no selective reporting or other bias. The results are presented in Figure 2.

### 3.4 The effects of different rTMS protocols on body structure, body function, and activity

#### 3.4.1 The effects of different rTMS protocols with FMA-UE

**Figure 3:**
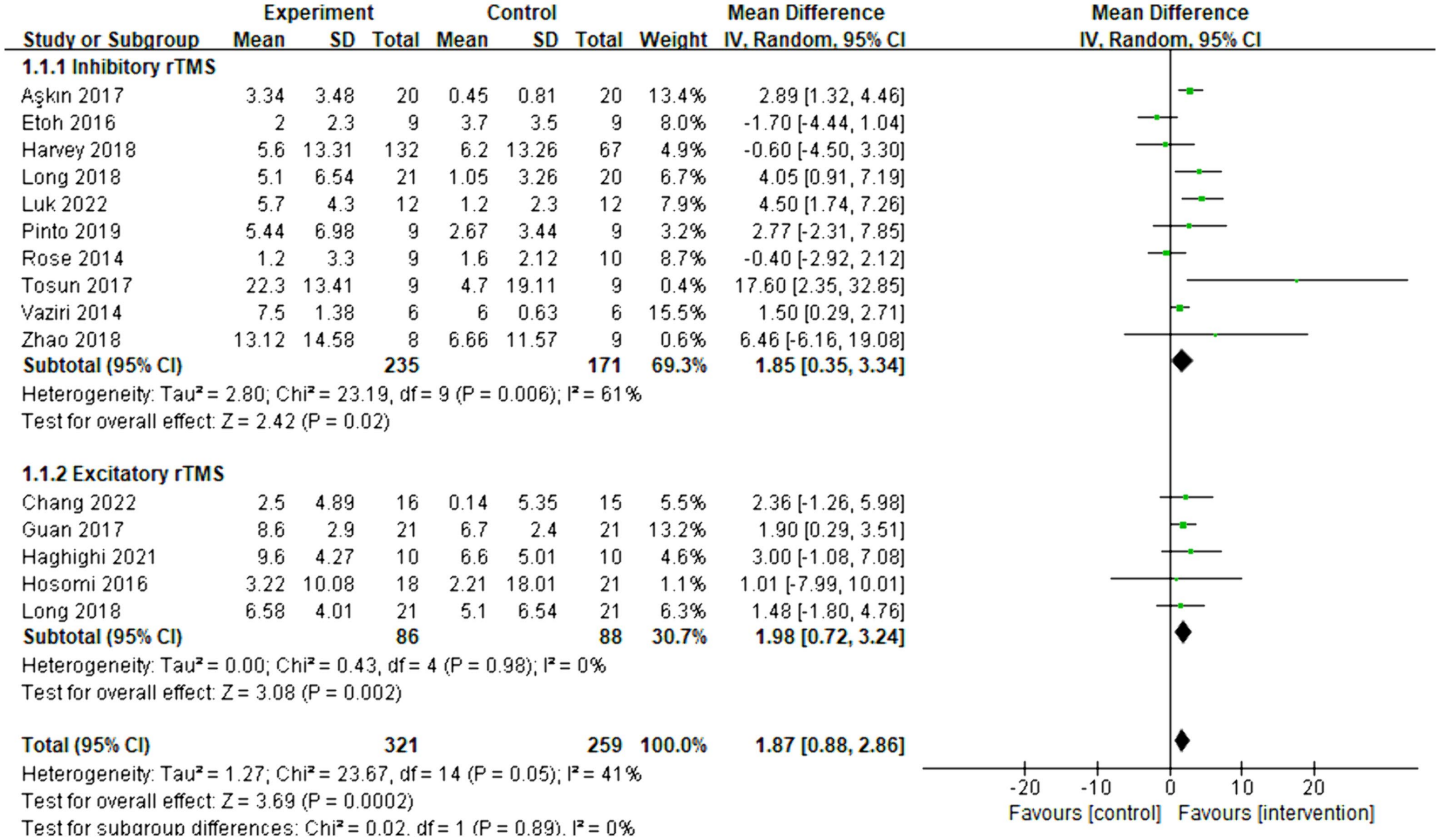
Forest plot of mean difference and 95% CIs for the effects of rTMS with FMA-UE. The abbreviation of CIs indicates Confidence Intervals; rTMS, repetitive Transcranial Magnetic Stimulation; and FMA-UE, Fugl Meyer assessment upper extremity scale.

Fourteen articles were included in the meta-analysis, and the behavioral improvement was evaluated using FMA-UE. The overall effect size (MD = 1.87, 95% CI = [0.88]–[2.86], *p* < 0.001) indicated a significant increase in favor of the intervention group. Subgroup analysis revealed that both inhibitory (MD = 1.85, 95% CI = [0.35]–[3.34], *p* = 0.02) and excitatory (MD = 1.98, 95% CI = [0.72]–[3.24], *p* = 0.002) rTMS protocols significantly facilitated an increase. The forest plot is presented in Figure 3. As shown in Supplementary Figure 1, the funnel plot was basically symmetrical.

#### 3.4.2 The effects of different rTMS protocols with Brunnstrom recovery stage

**Figure 4:**
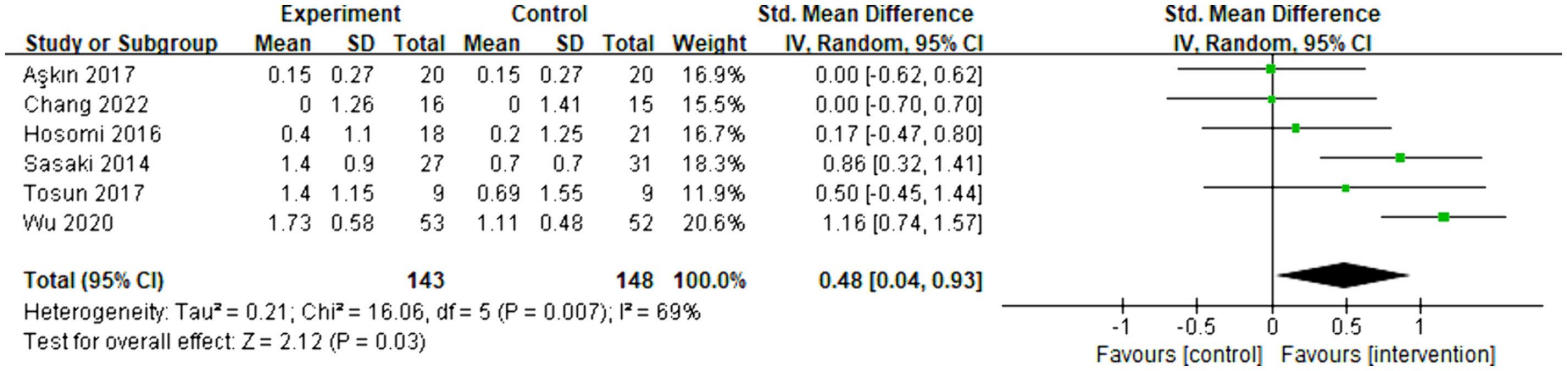
Forest plot of standard mean difference and 95% CIs for the overall effects of rTMS with Brunnstrom recovery stage. The abbreviation of CIs indicates Confidence Intervals; rTMS, repetitive Transcranial Magnetic Stimulation; Std. Mean difference, Standard mean difference.

Six articles were included in the meta-analysis, evaluating the behavioral improvement using the Brunnstrom recovery stage. The overall effect size (SMD = 0.48, 95% CI = [0.04]–[0.93], *p* = 0.03) indicated a significant increase in favor of the intervention group. However, due to the limited number of articles and the mixed rTMS protocol, conducting a subgroup analysis was temporarily unfeasible. The forest plot can be seen in Figure 4.

#### 3.4.3 The effects of different rTMS protocols with ARAT

**Figure 5:**
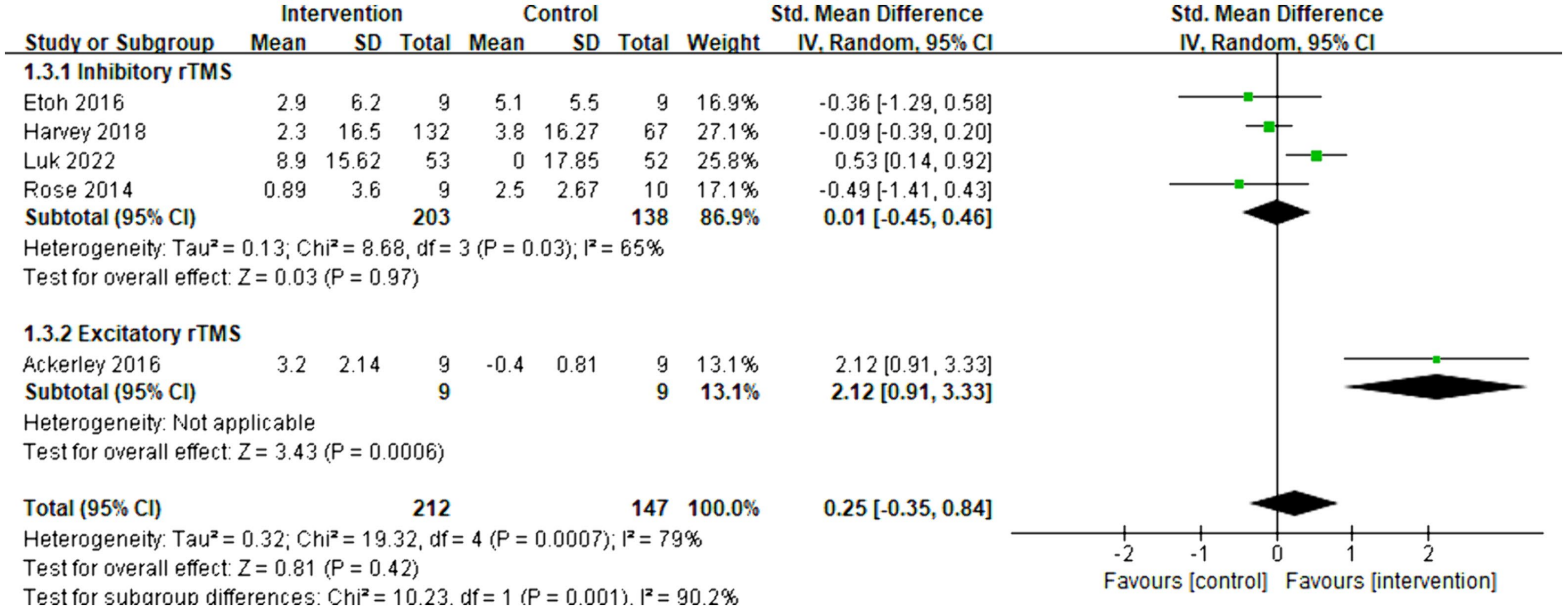
Forest plot of standard mean difference and 95% CIs for the effects of rTMS with ARAT. The abbreviation of CIs indicates Confidence Intervals; rTMS, repetitive Transcranial Magnetic Stimulation; ARAT, Action research arm test; and Std. Mean Difference, Standard mean difference.

The meta-analysis included five articles and evaluated the functional improvement using the ARAT. Overall, there was no significant increase observed between the control and intervention groups (SMD = 0.25, 95% CI = [−0.35]–[0.84], *p* = 0.42). Subgroup analysis revealed that inhibitory rTMS (SMD = 0.01, 95% CI = [−0.45]–[0.46], *p* = 0.97) did not have an effect on the ARAT score, whereas excitatory rTMS (SMD = 2.12, 95% CI = [0.91]–[3.33], *p* < 0.001) significantly changed the ARAT score. However, only one article related to excitatory rTMS was available. The forest plot is presented as Figure 5.

#### 3.4.4 The effects of different rTMS protocols with Barthel index

**Figure 6:**
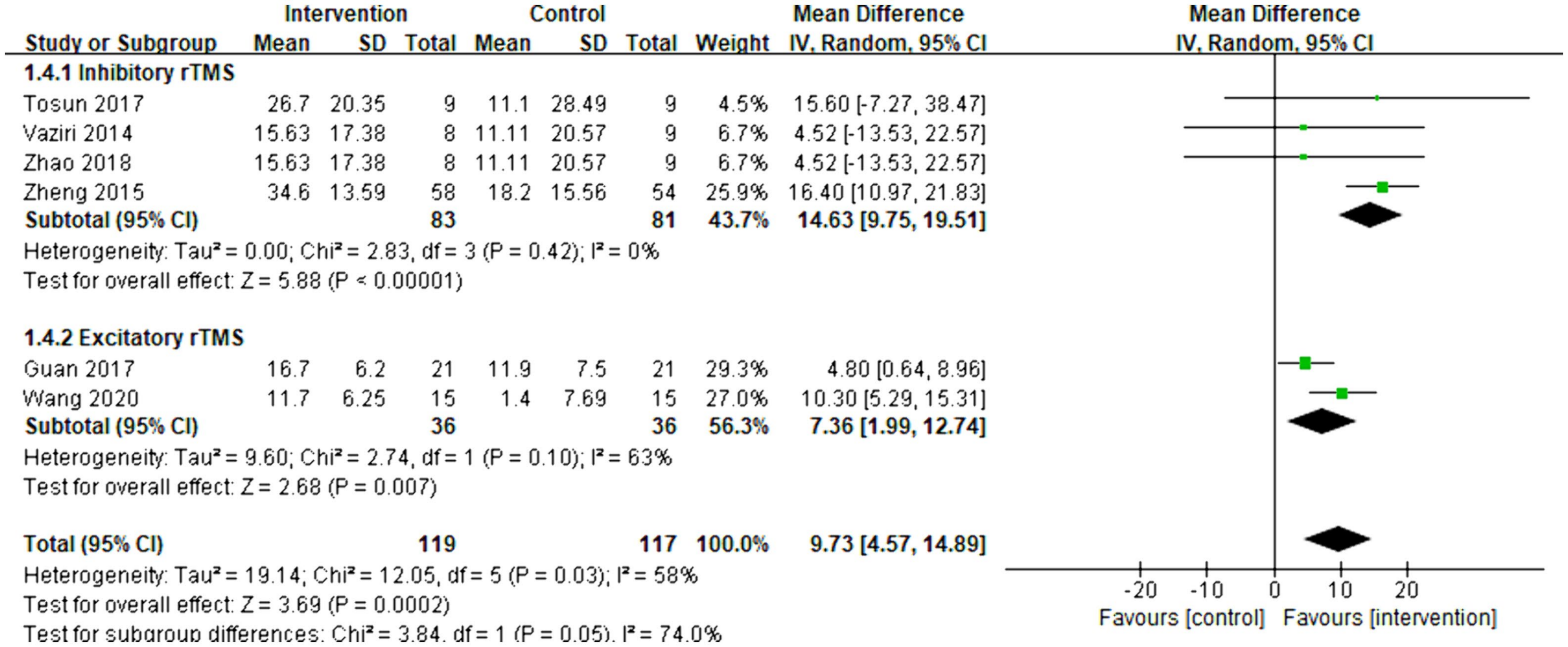
Forest plot of mean difference and 95% CIs for the effects of rTMS with Barthel index. The abbreviation of CIs indicates Confidence Intervals; rTMS, repetitive Transcranial Magnetic Stimulation.

Six articles were included in the meta-analysis. The improvement in daily life activities was assessed using the Barthel index. The overall effect size (MD = 9.73, 95% CI = [4.57]–[14.89], *p* < 0.001) indicated a significant increase in favor of the intervention group. Subgroup analysis demonstrated that both inhibitory (MD = 14.63, 95% CI = [9.75]–[19.51], *p* < 0.001) and excitatory rTMS (MD = 7.36, 95% CI = [1.99]–[12.74], *p* = 0.007) were significantly beneficial for the Barthel index. The forest plot is presented in Figure 6.

### 3.5 The effects of rTMS on different severities of hemiplegia and stroke stages

#### 3.5.1 The effects of rTMS on different severities of hemiplegia

**Figure 7:**
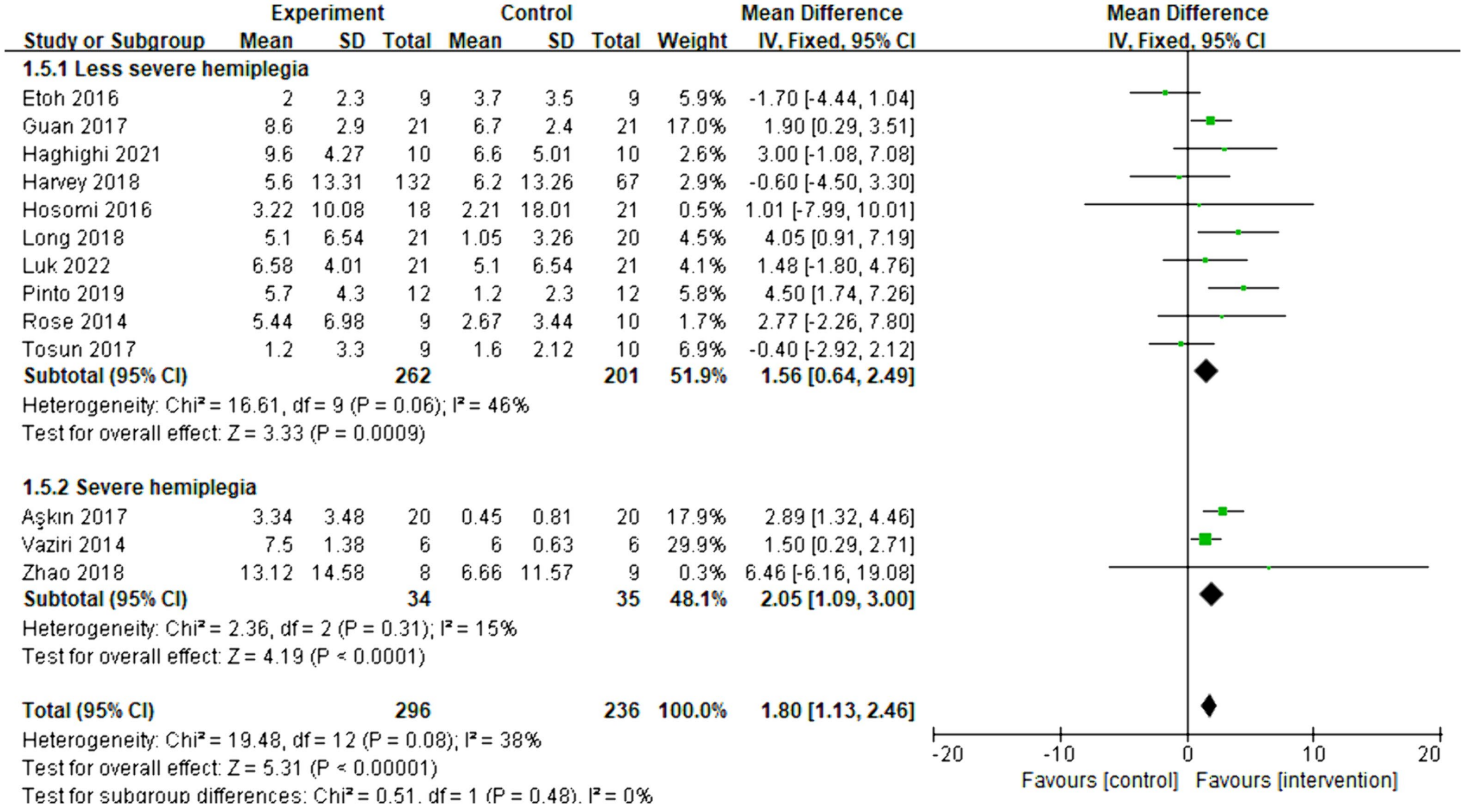
Forest plot of mean difference and 95% CIs for the effects of rTMS on different severities of hemiplegia based on FMA-UE. The abbreviation of CIs indicates Confidence Intervals; rTMS, repetitive Transcranial Magnetic Stimulation; and FMA-UE, Fugl Meyer assessment upper extremity scale.

Thirteen articles were included in the meta-analysis. The evaluation of behavior improvement was done using FMA-UE. The overall effect size (MD = 1.80, 95% CI = [1.13]–[2.46], *p* < 0.001) indicated a significant increase in favor of the intervention group. Furthermore, the relationship between the effect of rTMS and the severity of hemiplegia was examined. The subgroup analysis results revealed that rTMS was significantly effective for both less severe (MD = 1.56, 95% CI = [0.64]–[2.49], *p* < 0.001) and severe (MD = 2.05, 95% CI = [1.09]–[3.00], *p* < 0.001) hemiplegia. The forest plot is presented in Figure 7. As shown in Supplementary Figure 2, the funnel plot was basically symmetrical.

#### 3.5.2 The effects of different rTMS protocols on different severities of hemiplegia in acute and subacute stroke

**Figure 8:**
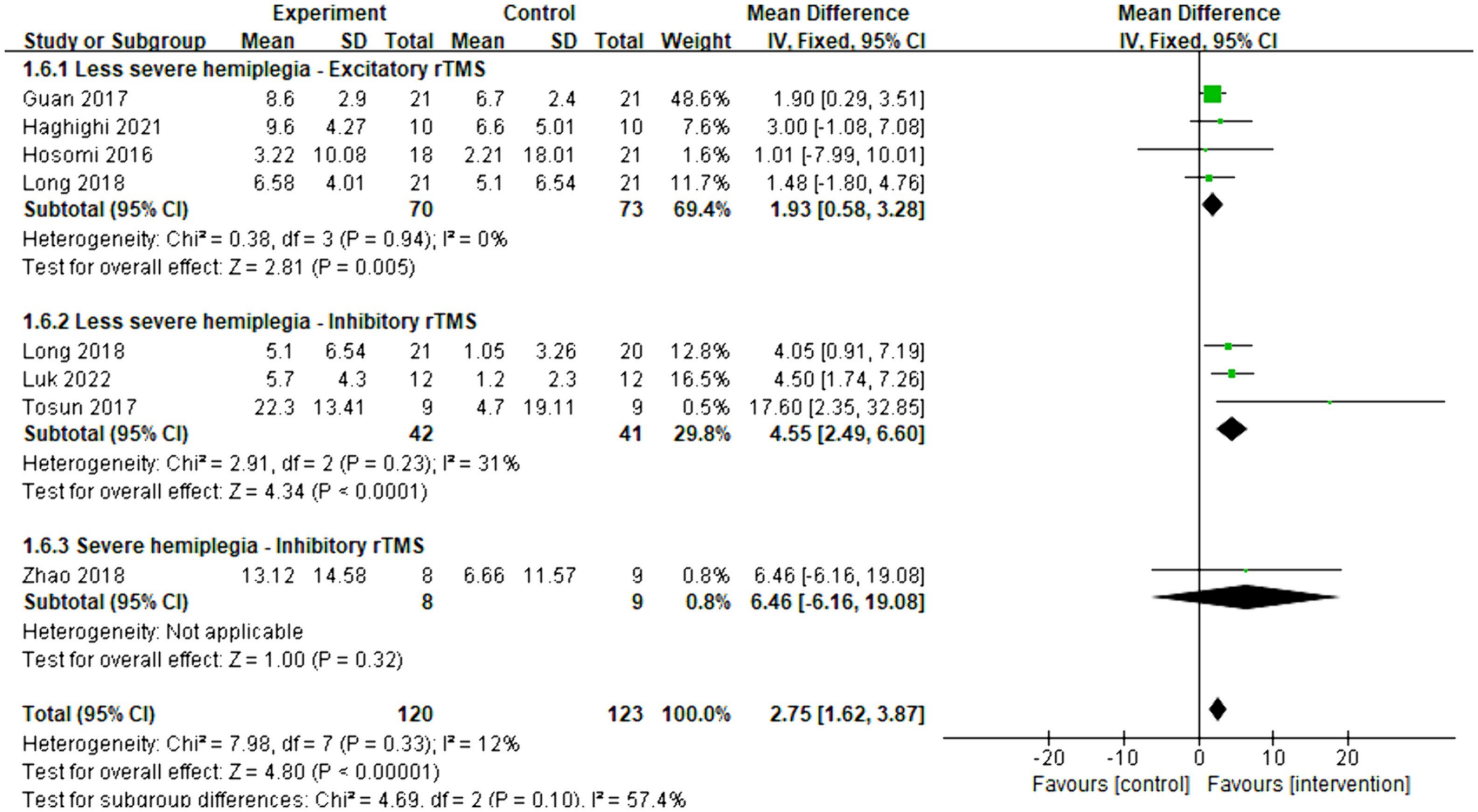
Forest plot of mean difference and 95% CIs for the effects of rTMS on different severities of hemiplegia in acute and subacute stroke based on FMA-UE. The abbreviation of CIs indicates Confidence Intervals; rTMS, repetitive Transcranial Magnetic Stimulation; and FMA-UE, Fugl Meyer assessment upper extremity scale.

Eight articles were included in the meta-analysis to evaluate the improvement in behavior using FMA-UE. The overall effect size (MD = 2.75, 95% CI = [1.62]–[3.87], *p* < 0.001) indicated a significant increase in favor of the intervention group. Subgroup analysis revealed that both excitatory (MD = 1.93, 95% CI = [0.58]–[3.28], *p* = 0.005) and inhibitory (MD = 4.55, 95% CI = [2.49]–[6.60], *p* < 0.001) rTMS were found to be beneficial for less severe hemiplegia. However, inhibitory rTMS (MD = 6.46, 95% CI = [−6.16]–[19.08], *p* = 0.32) appeared to be not significantly effective for severe hemiplegia. There were no articles discussing the effect of excitatory rTMS on severe hemiplegia in acute and subacute stroke. The forest plot is presented in Figure 8.

#### 3.5.3 The effects of different rTMS protocols on different severities of hemiplegia in chronic stroke

**Figure 9:**
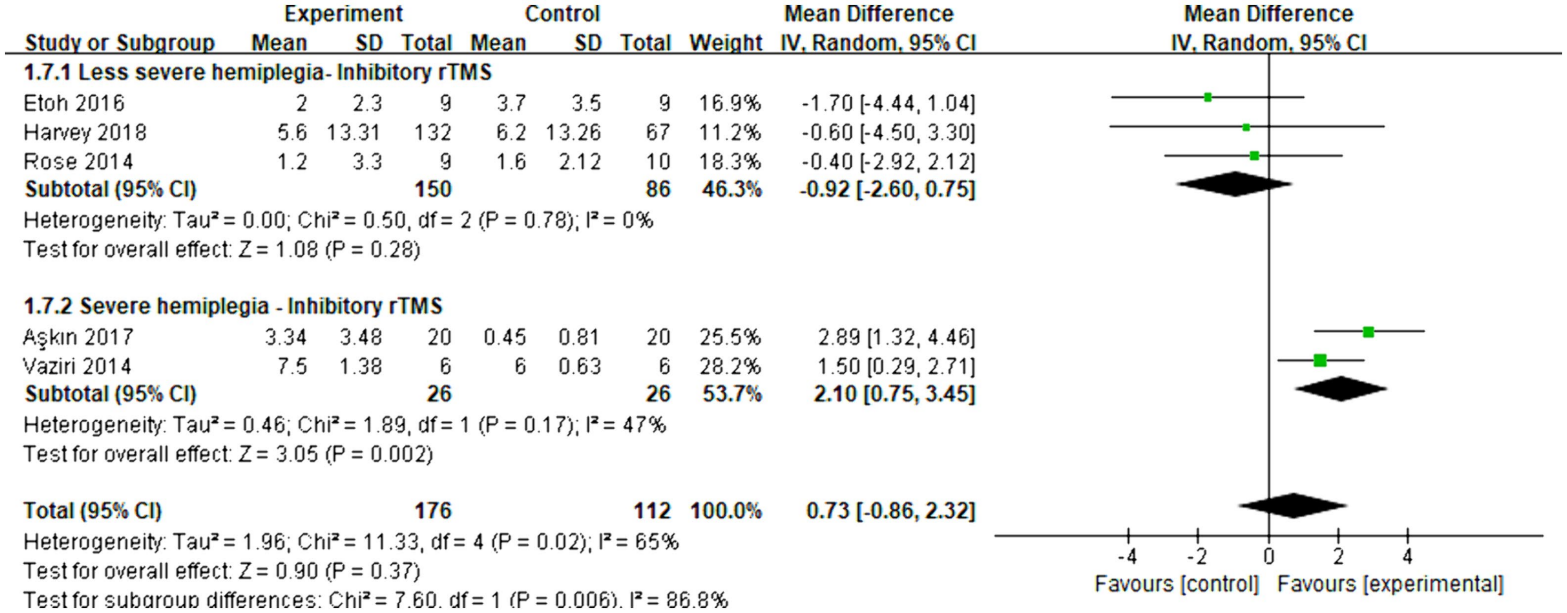
Forest plot of mean difference and 95% CIs for the effects of rTMS on different severities of hemiplegia in chronic stroke based on FMA-UE. The abbreviation of CIs indicates Confidence Intervals; rTMS, repetitive Transcranial Magnetic Stimulation; FMA-UE, Fugl Meyer assessment upper extremity scale.

Five articles were included in the meta-analysis to evaluate the improvement in behavior using FMA-UE. The overall effect size (MD = 0.73, 95% CI = [−0.86]–[2.32], *p* = 0.37) indicated no significant increase in favor of the intervention group. Subgroup analysis revealed that inhibitory rTMS (MD = -0.92, 95% CI = [−2.60]–[0.75], *p* = 0.28) did not provide benefits for less severe hemiplegia. However, it proved to be significantly effective in enhancing the behavior performance of stroke patients with severe hemiplegia (MD = 2.10, 95% CI = [0.75]–[3.45], *p* = 0.002). Figure 9 presents the forest plot.

## 4 Discussion

In this study, our meta-analysis investigated the effects of rTMS on FMA-UE, Brunnstrom recovery stage, Barthel index score, and ARAT score with 19 papers involving 865 patients. Our findings suggest that both inhibitory and excitatory rTMS can improve FMA-UE and Barthel index scores; rTMS is beneficial for both less severe and severe hemiplegia unless stratified analysis is performed. Inhibitory rTMS was significantly effective in non-chronic stroke but not in chronic stroke for less severe hemiplegia, whereas it was not significantly effective in non-chronic stroke but significantly effective in chronic stroke for severe hemiplegia. On the other hand, excitatory rTMS was found to be significantly effective in non-chronic stroke for less severe hemiplegia. These findings are crucial in guiding the selection of rTMS parameters for upper limb hemiplegia resulting from a stroke. Importantly, this study is the first to simultaneously investigate the effects of rTMS based on stroke stage, hemiplegia severity, and rTMS parameters. The stratified effects of rTMS on the improvements in Brunnstrom recovery stage and ARAT need further research.

### 4.1 The overall effects of rTMS on body function and activity

All rTMS interventions demonstrated a positive impact on upper limb motor function, as assessed by the FMA-UE. However, when the ARAT was used as a measure, the advantages of rTMS were no longer evident. This discrepancy can be attributed to the fact that the FMA-UE assesses functional abilities, whereas the ARAT evaluates activity level. Therefore, it is possible that rTMS improves motor function but does not have the same effect on activity level. This finding is consistent with a previous study on bilateral arm training, which reported significant improvements in FMA-UE scores but less significant improvements in ARAT scores (40). The difference in results between the ARAT and FMA-UE may be due to the stronger floor effect of the ARAT, which requires precise coordination of multiple components (shoulder, elbow, forearm, and hand), while the FMA-UE only requires partial integration (41, 42). The integration of upper limb movement is not only dependent on the M1, but also on other motor-related areas. However, most rTMS stimulation sites currently focus on the M1 of both hemispheres. This suggests that we should consider extending our focus to other motor-related areas.

Additionally, rTMS has the potential to improve the Barthel index, which includes both upper limb-related and lower limb-related movements. We hypothesized that the relationship between improved Barthel index and upper limb motor function is currently unknown. As there are not enough inhibitory and excitatory rTMS studies about Brunnstrom recovery stage, it needs further research.

### 4.2 The effects of rTMS on different severities of hemiplegia and stroke stages

We found that both severe and less severe hemiplegia benefited from rTMS. This indicates that rTMS is generally useful, similar to the brain computer interface, which is also effective in promoting improvements in stroke patients with mild, moderate, and severe upper limb impairment (43). However, it is evident that the one size fits all theory needs improvement. Stroke patients with different levels of motor impairment exhibit varying upper limb use patterns (44), use duration, and laterality preferences (45). They even have different white matter microstructures. For instance, the corticospinal tract asymmetry index is associated with all levels of upper limb impairment, while corpus callosum microstructure is more suitable for explaining severe upper limb impairment post-stroke (46). Additionally, the potential for recovery varies between chronic and non-chronic stroke cases. The optimal recovery period is within 3 months after stroke, particularly within 4 weeks of its onset (47). Therefore, when selecting rTMS parameters, it is crucial to consider the different stroke stages and hemiplegia severities in order to develop individualized rehabilitation programs in clinical practice. Stratifying stroke patients based on stroke stage and hemiplegia severity is imperative. A study has already attempted to stratify chronic stroke patients based on the role of the contralesional hemisphere (48). However, there is currently no detailed stratifying study on rTMS therapy.

### 4.3 The effects of inhibitory rTMS based on stroke phase and hemiplegia severity

Our findings indicated that inhibitory rTMS was significantly effective for less severe hemiplegia in the acute and subacute phases, but not in the chronic phase. However, inhibitory rTMS was not significantly effective for severe hemiplegia in the acute and subacute phases, but significantly effective in the chronic phase. This stratification result presents a significant challenge and deserves careful consideration.

Previous studies have provided support for the concept of a time window in rehabilitation therapy. Since rehabilitation relies on brain plasticity, it is important to note that brain plasticity also has its own time window. Rapid functional improvement is typically observed within this time window (2). For example, Hordacre et al. conducted a study to investigate the inhibitory effect of continuous theta-burst stimulation over the contralesional hemisphere in poststroke patients. The results showed that this effect was strongest at 2 weeks poststroke and gradually weakened over time (49). Additionally, Everard et al. found that virtual reality and robot assistant technology were more advantageous during the subacute phase of stroke, whereas all rehabilitation technologies appeared to be equally effective during the chronic phase (50). These findings highlight the importance of identifying the optimal time window for intervention based on the specific features of hemiplegia, in order to facilitate maximum recovery.

The impact of inhibitory rTMS on severe and less severe hemiplegia can yield completely opposite results. It is generally accepted that recovery in the ipsilesional hemisphere indicates a good prognosis, while recovery in the contralesional hemisphere suggests a poor prognosis (51). Previous studies have suggested that inhibiting the contralesional hemisphere with inhibitory rTMS could be an effective approach to restore function in the ipsilesional hemisphere, based on the interhemisphere inhibition theory (52). However, further research has provided new insights into interhemispheric inhibition (53). The role of the contralesional hemisphere can vary depending on the severity of hemiplegia. In cases of severe hemiplegia, there is a decrease in interhemispheric inhibition of the contralesional hemisphere as motor impairment increases. Conversely, in cases of less severe hemiplegia, there is an increase in interhemispheric inhibition of the contralesional hemisphere as motor impairment increases (48). This suggests that the contralesional hemisphere may provide support when motor impairment is extremely mild or severe, while moderate motor impairment exhibits greater inter-hemispheric inhibition. Carson et al. even came up with the opinion that inter-hemisphere inhibition model arose from a fundamental misunderstanding of the physiological properties exhibited by inter-hemispheric projections. Instead of just preventing over-excitation, the inhibitory interneurons tried to sculpt the output of specific circuits (54). Therefore, the inter-hemisphere relationship is variable.

Based on our hypothesis that brain plasticity starts early in patients with less severe hemiplegic stroke, resulting in significant interhemispheric inhibition, we conducted a meta-analysis. As a result, inhibitory rTMS was found to be significantly effective for less severe hemiplegia during the acute and subacute phases. However, during the chronic phase, it was no longer a suitable time window for remodeling brain function. Therefore, inhibitory rTMS was not significantly effective for less severe hemiplegia during the chronic phase. On the other hand, initiating brain plasticity was challenging for severe hemiplegia due to the extent of brain damage. Consequently, inhibiting the contralesional hemisphere was found not to result in significant improvement. A study supported our hypothesis, showing no significant improvement in FMA and Barthel index scores after low-frequency rTMS over M1 of the contralesional hemisphere for severe hemiplegia within 2 weeks to 3 months after stroke. Interestingly, stroke patients showed greater improvement in the FMA and Barthel index scores after high-frequency rTMS over M1 of the contralesional hemisphere (36). It was only during the chronic phase that brain plasticity gradually recovered for stroke patients with severe hemiplegia. Consequently, the effect of inhibitory rTMS could be observed during the chronic phase in severe hemiplegia patients.

To conclude, the role of the contralesional hemisphere in chronic stroke remains unresolved (55). Further high-quality stratified research is urgently needed to elucidate the role of the dynamic contralesional hemisphere.

### 4.4 The effect of excitatory rTMS based on stroke phase and hemiplegia severity

As mentioned above, ipsilesional hemisphere recovery is associated with a positive prognosis (51). Excitatory rTMS can directly target the affected hemisphere. Therefore, we hypothesized that excitatory rTMS would achieve a more significant effect than inhibitory rTMS. A meta-analysis (56) and a network meta-analysis (57) have indeed confirmed that high-frequency rTMS was superior to low-frequency rTMS, although the number of studies on the former was relatively small.

According to our study, excitatory rTMS was found to be significantly effective in the acute and subacute phases of less severe hemiplegia, which is consistent with a previous study that demonstrated the benefits of both low-frequency and high-frequency rTMS in motor recovery among stroke patients (58). Our results also support the interhemisphere inhibition theory. However, there is a lack of available meta-statistics on the use of excitatory rTMS for less severe hemiplegia during the chronic phase of stroke, possibly due to inconsistent evaluation indicators that do not meet our statistical requirements.

Regarding severe hemiplegia, no studies were found on the effectiveness of excitatory rTMS. However, a resting state fMRI study indicated that stroke patients with lower baseline functional connectivity of bilateral M1 may benefit more from high-frequency rTMS (58). Additionally, stimulating the premotor cortex with transcranial direct current stimulation has been shown to improve inter-hemisphere functional connectivity in moderate-to-severe chronic stroke (59). On the other hand, a different study suggested that rTMS may be less effective for severe motor impairment (2). We believe this could be due to the presence of cortical injury often associated with severe hemiplegia. Directly stimulating the injured cerebral cortex is considered a potential risk factor for epilepsy, which is why researchers approach the use of excitatory rTMS with caution.

### 4.5 Limitations

Some limitations of our study should be concerned. First, although we have searched the database thoroughly, there is a shortage of relevant studies about severe hemiplegia and excitatory rTMS. Some subgroups contain only one or two studies. This gives us a hint that we can pay more attention to these issues in future research to obtain more robust conclusion. Second, when doing subgroup analysis, some studies were not included because the severity of stroke was not mentioned. This may have a certain impact on the results more or less. It suggests that our inclusion criteria should be clear enough in the future research. Third, high heterogeneity exists among studies of inhibitory rTMS. Subgroup analysis based on stroke staging and severity improved homogeneity. This indicates that these are the issues that we need to consider comprehensively when selecting rTMS parameters. The rTMS effect should be different between chronic and acute/subacute phase stroke, severe and less-severe stroke. Finally, our study revealed significant *p* values with relatively small MD in some instances when compared to the minimal clinically important difference values for the FMA-UE. This could be attributed to the large number of cases included in our study and the concentrated distribution of data. Nonetheless, it underscores the importance of meticulous calculation and application of the minimal clinically important difference in clinical research.

## 5 Conclusion

In our study, we conducted a detailed analysis of the rTMS parameters and its relationship with stroke patients. As demonstrated for the first time by our meta-analysis, the same rTMS parameters can have varying effects depending on the severity of hemiplegia and the stage of stroke. Therefore, when selecting the appropriate rTMS parameters, it is essential to take into account both the motor impairment and stroke stage. The development of a more effective stratification method for stroke patients depends on gaining a deeper understanding of the interhemisphere relationship. Further research can be conducted in this area in the future.

## Data availability statement

The original contributions presented in the study are included in the article/Supplementary material; further inquiries can be directed to the corresponding author.

## Ethics statement

Ethical approval was not required for the study involving humans in accordance with the local legislation and institutional requirements. Written informed consent to participate in this study was not required from the participants or the participants’ legal guardians/next of kin in accordance with the national legislation and the institutional requirements.

## Author contributions

RL: Conceptualization, Data curation, Formal Analysis, Funding acquisition, Methodology, Software, Supervision, Writing – original draft, Writing – review & editing. SL: Data curation, Writing – original draft. TL: Data curation, Writing – original draft. KY: Formal Analysis, Methodology, Software, Writing – original draft. XW: Investigation, Methodology, Resources, Software, Writing – original draft. WW: Investigation, Methodology, Resources, Software, Writing – original draft.
