# Supplementary figures and images for: The stratified effects of repetitive transcranial magnetic stimulation in upper limb motor impairment recovery after stroke: a meta-analysis
Source: Front Neurol. 2024 Apr 2;15:1369836. doi: 10.3389/fneur.2024.1369836 (PMC11020108; doi:10.3389/fneur.2024.1369836)

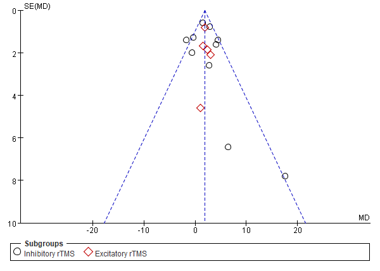

Supplement: Supplementary file 1 [file Image_1.TIF]

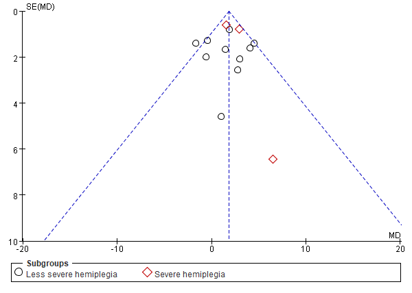

Supplement: Supplementary file 2 [file Image_2.TIF]
